# Microclimate of Brown Bear (*Ursus arctos* L.) Dens and Denning Area

**DOI:** 10.3390/ani14213124

**Published:** 2024-10-30

**Authors:** Damir Ugarković, Nikolina Kelava Ugarković

**Affiliations:** 1 Faculty of Forestry and Wood Technology, Department of Forestry, Institute of Ecology and Silviculture, University of Zagreb, Svetošimunska Cesta 23, 10000 Zagreb, Croatia; dugarkovic@sumfak.unizg.hr; 2 Faculty of Agriculture, Division of Animal Science, Department of Animal Science and Technology, University of Zagreb, Svetošimunska Cesta 25, 10000 Zagreb, Croatia

**Keywords:** air temperature, relative humidity, size, status, exposure, den, denning area, brown bear

## Abstract

The aim of this study was to determine the microclimate of brown bear dens depending on their size, their status (active or inactive), and the location of the den. The study included five dens of different sizes and locations in the Velebit Mountains, in the Dinaric Alps. The measurements of air temperature (°C) and relative humidity (%) in dens and the forest stands were carried out over 182 days. The absolute minimum air temperature in inactive dens was between −2.88 °C and −0.38 °C and belonged to the dens with shorter tunnels and without chambers. The exception was inactive den 1, which was situated in a thermophilic forest. No negative absolute minimum air temperatures were recorded in active dens. The absolute minimum air temperatures in the forest stands were lower than in the dens. The relative humidity was significantly higher in all dens than in the forest stands. Dens with a large entrance opening and a short tunnel were strongly influenced by the local microclimate of the forest stand. The greatest temperature difference (den–forest) was between elongated dens with chambers and the associated forest stand, while the smallest temperature differences occurred in dens with a short tunnel, without a chamber, and at the northern exposure. The greatest differences in relative humidity (den–forest) were found in dens with chambers. The den temperatures correlated with the air temperatures in the forest stands.

## Simple Summary

The den serves to protect bears from disturbances and harsh weather conditions during their hibernation. However, there is limited information about the microclimatic conditions within the den and the factors that influence them. We examined how temperature (°C) and relative humidity (%) in the den vary in relation to the microclimatic conditions in the surrounding forest stand and the den’s morphology. Our findings indicate that the characteristics of the forest stand affect the temperature within the den, while the size (small or large) and status (active or inactive) of the den influence both microclimatic parameters. When interpreting the microclimatic conditions, it is essential to consider the morphology of the den. Future research utilizing camera traps and a larger number of dens is necessary to validate these findings.

## 1. Introduction

The habitats of brown bears in the Dinaric Alps are considered suitable for the growth and stability of the population [1,2]. In this high karst area, the brown bear, as the largest wild animal, lives, feeds, reproduces, and dens. At the beginning of winter, bears retreat to remote and quiet forest areas that are difficult to access. Bear dens are usually located at higher altitudes, on sloping terrain and on the upper third of a slope [3,4]. In the Croatian Dinaric Alps denning area of brown bears is characterized by extremely rocky terrain with mainly beech–fir forests, mountain beech forests, and pre-mountain beech forests. Coastal beech forests are also found in somewhat more thermophilic areas [5].

The availability of safe places, i.e., nesting or denning areas, may be important for increasing the bear population and survival and minimizing energy consumption [6]. A bear den can be located in a small cave in a rock, in a tree root cavity, in a standing hollow tree or tree stump, in a hollow lying log, or an excavated space under a rock [2,7,8,9,10]. In the Dinaric Alps in Slovenia, most bear dens are karst caves, followed by rock shelters, and the fewest dens are open-air nests [11]. In the Velebit Mountains, in the Dinaric Alps in Croatia, most dens are rock cavities and excavations under rocks, and the fewest are hollow trees or tree stumps, hollow logs and ground nests [5].

The function of a bear’s den is to reduce energy loss and provide protection from disturbance in winter. Energy storage or conservation is ensured by the den’s insulation [12]. Different types of dens insulate bears differently and can affect heat loss, the amount of body fat in bears and the maintenance of body temperature [7]. The body temperature of a brown bear drops by 3 to 5 °C during hibernation compared to the temperature of an active bear that does not hibernate [13]. Choosing a quiet and safe place for denning is crucial for females, who give birth to their cubs in the den [11,14,15]. An inadequately prepared den, poor den selection or disturbance of the female bear during her stay can lead to reduced growth and increased mortality of offspring [16].

Only a few researchers have reported on the environmental conditions in the den area and in the den itself [17]. Huber and Roth [15] studied the denning of brown bears in the Republic of Croatia in the area of Gorski Kotar and the Plitvice Lakes, and Ugarković et al. [5,18] studied the morphological and relief features of dens and the denning ecology of the dens in the Velebit area. However, there have been no microclimatic studies in the denning area and the dens themselves in the Dinaric Alps area in Croatia.

Microclimatic conditions influence the survival and development of individuals within populations [19], meaning that the microclimate has a significant influence on population stability [20]. As the den plays an important role in the life of the brown bear, especially for pregnant females and newborn cubs, it is necessary to better understand its environmental factors, i.e., the microclimate condition of the den and denning area in winter. This study aimed to determine the microclimatic conditions in the dens concerning the size of the den, the status of the den (active or inactive, i.e., hibernating or not hibernating), and the microclimate of the denning area.

## 2. Materials and Methods

### 2.1. Research Area

**Figure 1:**
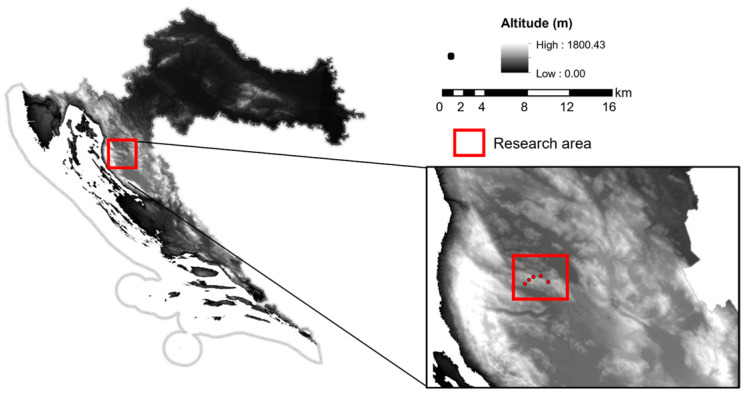
Map of the Republic of Croatia with research area marked.

The brown bear dens included in the research were located in the high karst. All the elements and phenomena characteristic of the karst can be found there: peaks, heads, hips, sinkholes, depressions and valleys. According to Köppen, the climate of the studied area belongs to classes C and D, types Cf and Df [21]. The study was conducted in the Linarski vrh (den 1: 5,517,428, 4,961,662), Crni vrh (den 2: 5,517,484, 4,961,637), Bilo (den 3: 5,516,240, 4,960,800 and den 4: 5,521,528, 4,960,318), and Korenski vrh (den 5: 5,519,480, 4,961,960) areas (Figure 1). These micro-sites of the research area covered 1 km^2^. In addition, maps of hunting grounds and hunting chronicles were analyzed to determine the forest areas where bears den. In addition, based on previous research on brown bear ecology and population management [1,15,22,23], areas were selected that provide denning conditions and are favored by brown bears.

### 2.2. Measurements of Microclimate and Description of Dens and Denning Area

The microclimatic conditions were measured with an iButton measuring device (iButtonLink Technology, Whitewater, WI, USA). This device measures air temperature (°C) and relative humidity (%). The device was protected, i.e., it was placed in a protective shield so that it was not in contact with the rocks or the ground in the den. A bear den mainly consists of three parts, namely an entrance, a tunnel, and a chamber with a nest made of twigs, leaves, moss, and other material [17]. The measuring device was placed in the tunnel of the den at least 100 to 150 cm from the end of the den.

In addition, a measuring device was set up in the forest stand near the den, about 25 m from the den entrance. The device was attached to a tree at a height of 1.5 to 2 m. All the devices in the dens and the forest stands were synchronized and simultaneously measured the microclimatic conditions. The microclimatic conditions were measured hourly, starting on 3 November 2023, and ending on 2 May 2024. Denning in the study area begins in the late autumn with a mean hibernation of 94 days and lasts until 11 March [11]. After reading the values of the climatic elements, daily averages were calculated, covering 182 days. This was a longer period than the average hibernation or stay in the den. In the Dinaric Alps, male bears hibernate for an average of 57 days (range from 17 to 90 days) and female bears an average of 82 days (range from 10 to 142 days) [11].

Altitude (GPSMAP^®^ 65s, Olathe, KS, USA, SAD; m above sea level), slope (Suunto Tandem/360PC/360R G Clino, Vantaa, Finland; %) and exposure (GPSMAP^®^ 65s, Olathe, KS, USA, SAD; azimuth °) were measured at the entrance of the den. The dimensions (width, height) of the den were measured every 100 cm using a tape measure and a Leica rangefinder (Leica, Wetzlar, Germany). The den’s dimension data were processed using the Compass Cave software package (https://fountainware.com/compass/, (accessed on 28 October 2024)) [24]. The size of the den was determined by volume (m^3^). Dens with a volume of up to 5 m^3^ were considered small, and those with a volume > 5 m^3^ were considered big.

Table 1 lists information on each den included in the study. Several limiting factors (relief factors, size of the study area, rockiness, and habitat conditions) prevented the inclusion of more dens and dens of different size or status in the study. In addition, the study area was located within a hunting ground concession and permits needed to be obtained to set up measuring devices in the dens. The status of the den (active or inactive) was determined based on the changes observed in the den in May 2024 compared to the situation in November 2023. These included traces of bear hair, paw prints, and the condition of the bedding material in the den. As no monitoring with cameras (photo traps) was carried out and the dens were not visited during the study period to minimize disturbance of the bears, no precise information is available on the exact duration of the bears’ stay in the den or on the status of the bears (sex, age, pregnant female, female with cubs). Forest ecosystems in the study area were characterized as thermophilic (black hornbeam forests) and mesotermophilic (beech–fir forests) [5].

**Figure 2:**
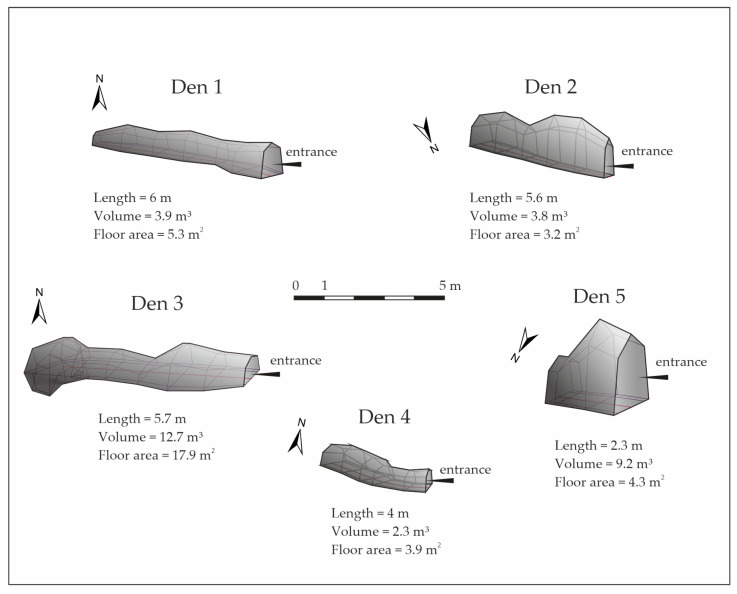
3D models and shapes of analyzed brown bear dens.

The schematic representations (3D model) of the dens in the study area are shown in Figure 2.

### 2.3. Statistical Analysis

The comparison of microclimatic conditions in the den and the control area in the forest was carried out using Student’s *t*-test for dependent samples. Analysis of variance (ANOVA) was used to compare daily differences in air temperature and relative humidity (den minus control—forest). The factors were den size (volume), den status (active or inactive), and exposure. Factorial ANOVA was performed to analyze temperature and relative humidity differences depending on den size and status. A non-parametric Pearson correlation analysis was performed for the microclimatic conditions of all dens and all control plots in the forest. The significance level for all analyses was *p* < 0.05. Statistical data processing was performed using the Statistics v13 program [25].

## 3. Results

A total of six brown bear dens were found in the study area, five cave dens and one rock shelter. The absence of other den types such as hollow trees, tree stumps, or hollow logs can be attributed to the intensive forestry and selective forest management in the study area [5]. No burrows or root cavities dug into the ground were found either, as the ground in the study area is very shallow [26]. When bears choose a place to make a den in the ground, the type of soil is of crucial importance [27]. In a previous study, only 3% of dens reported in the Velebit Mountains were root excavation dens, while cave dens in this region with rocky terrain accounted for 77% of all dens [5]. The dens studied in this research were tubular cave forms such as holes in rocks, dens with a long tunnel and a small chamber at the end of the tunnel, and dens with short tunnels. Petram et al. [28] found that bears use many types of dens in rocky areas that are rarely visited by humans, but prefer deep caves or caves with small openings in areas that are more accessible to humans.

A smaller number of active dens was found than expected. There are various reasons why bears visit and use dens, including reduced food supply, snowfall, low temperatures, and physical condition [7,29,30,31]. Many authors [32,33,34] have reported that snowfall stimulates denning, but there are also some studies reporting the opposite [3,35,36]. However, the winter during the study period was very mild, there were only 14 days with snowfall, and the maximum snow depth was 20 cm [37]. This can be considered as one of the reasons why only two (40%) of the five dens were active during winter. In the present study, only one den was south-facing while south and west exposures are more typical for the northern areas of the Far East, Yakutia, the Volga–Kama region, and the Carpathians [38]. Thus, only about 25% of the dens in the Velebit have a southern exposure [18].

The microclimate measurements were successfully carried out in five dens, while the measuring device was lost in one den, where it was presumably found by a brown bear and removed from the den. Traces of a brown bear were found in this cave, but the measuring device was not found.

### 3.1. Microclimate Conditions in Dens and Denning Area

Table 2 contains descriptive statistics of air temperature (°C) and relative humidity in the studied dens and the corresponding forest stands as control sites. In the dens, the absolute minimum air temperatures ranged between −2.88 °C and 5.99 °C. In dens 4 and 5, the lowest absolute minimum air temperatures were measured, −2.88 °C and −0.38 °C, respectively. Den 4 had a short tunnel and no chamber, while den 5 was a rock shelter. Both dens were inactive during the winter months, i.e., no traces of bear activity were observed. No negative absolute minimum air temperatures were measured in the other dens. Dens with longer tunnels and chambers at the end of the tunnel had higher minimum temperatures (den 1 and den 3). The average air temperatures in the dens were in the positive range between 4.36 and 7.89 °C during the entire study period. The absolute minimum air temperatures in the corresponding forest stands were between −7.62 °C and −8.98 °C. In the past, an absolute minimum air temperature of −33.1 °C has been measured and reported in this area [39].

The microclimatic measurements show that the winter was warm during the study period, which is a consequence of the fluctuations in climatic elements and global warming [40]. Changes in the main climatic elements in the Velebit Mountains, as part of the Dinaric Alps, are also ongoing. A relatively warm late fall, winter, and early spring was recorded in the study area, as shown by the average air temperatures of the denning area (from 4.67 °C to 5.86 °C) and the absolute minimum (−8.98 °C) and maximum (18.8 °C) air temperatures. Rising temperatures influence the food availability and habitat preferences of brown bears [41,42]. According to studies by Acarer [43], the current habitats of brown bears in Europe will change regionally under the influence of climate change. The same author has reported that, according to various scenarios of climate change in Europe up to the year 2100, the habitats of brown bears will be reduced or fragmented or disappear completely in some regions.

The relative humidity in the dens ranged from 64.68% to 100% (Table 2). The absolute lowest relative humidity was measured in den 5, which has a large entrance opening and a very short tunnel (rock shelter) and therefore is strongly influenced by the microclimate of the forest stand. The absolute minimum relative humidity in the forest stands ranged from 21.72% to 45.36%. The lowest relative humidity in a den was found in the black hornbeam forest ecosystem (thermophilic) on the south exposure. These are more thermophilic forests [44] with more sun insulation, and since air temperature is negatively correlated with humidity, the stands have the lowest values of relative humidity [45,46,47]. The absolute maximum of relative humidity was 100%, corresponding to the per-humid climate in the study area [21].

According to Mažar [41], trends in mean annual and absolute maximum air temperatures have increased significantly from 1961 to 2013. Trends in the number of warm and hot days have also increased significantly, while the climate trend in the amount of precipitation has decreased. According to Tikvić et al. [42], there has been a significant increasing trend in air temperature at the Velebit meteorological stations, which is also the reason for the low number of days with snow in the Velebit region. A warmer climate leads to a decrease in snow cover [48]. Snow cover is the most sensitive to changes in temperature and precipitation, which is why it is considered a good indicator of climate change. According to IPCC [49], the temperature will rise faster in winter than in the other seasons (by more than 1 °C), especially in the distant future (2071–2100) [50]. A significant decreasing trend in the number of days with snow and the maximum snow depths has been reported in the Velebit area (1594 m above sea level, foothill vegetation zone) [51]. Monitoring changes in the bear habitat is necessary to recognize and implement conservation measures [22]. It is, therefore, necessary to continue monitoring not only the macroclimate but also the microclimate in the denning area.

**Figure 3:**
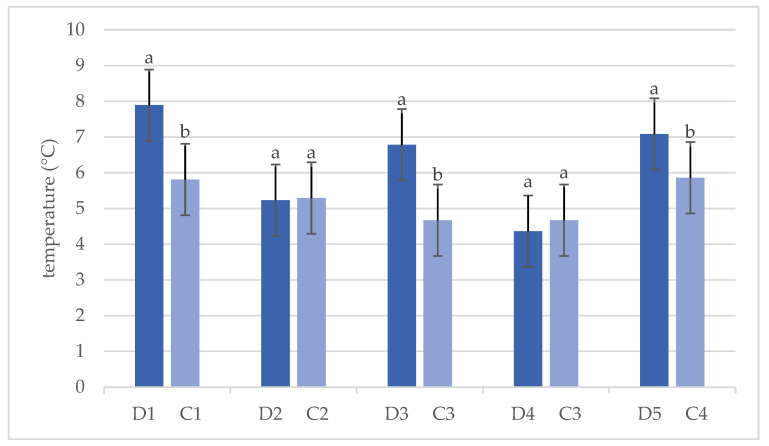
Results of Student’s *t*-test for the air temperatures (°C) in dens (D) and corresponding forest stands (C). (Bars represent average ± standard deviation. Values marked with a different letter are significantly different at level *p* < 0.05).

Figure 3 shows the results of the Student’s *t*-test for the air temperatures in the dens and their controls. The average air temperatures in dens 1, 3, and 5 were higher (*p* < 0.0001) than in the corresponding forest stands, while there was no difference in air temperatures between dens 2 and 4 and the corresponding forest stands. In addition, air temperatures were slightly higher in the forest stands than in the dens. Both dens (2 and 4) were small cave dens, i.e., dens with smaller dimensions, shorter overall length, and short tunnels. It can be assumed that these temperature differences would be greater if there were snowy conditions, i.e., if there was a lot of snow in winter. This means that the snow covers the den entrance or that the den entrance remains open in the upper third or upper quarter. This creates a specific microclimate in the den, especially when the bear is hibernating. The bear therefore plays an active role in shaping the microclimate in the den [52]. However, the maximum snow depth during the study period was 20 cm [37], which is insufficient considering the sizes of the entrances to these dens. Den 2 had an entrance height of 1.4 m, while den 4 had a large entrance width of 90 cm. In the past, maximum snow depths in the study area have ranged from 156.5 cm to 220 cm [39], and depths of 60.09 cm and 165.04 cm were also measured in 2021 [51]. This means that, if these dens have a snow-covered entrance in winters with high snow depths, a different microclimate is created. These results are in contrast to the findings of Craighead and Craighead [33] and Petram et al. [28], who reported that the small size of the den compared to the size of the bear allows for greater thermal stability, which is particularly evident in excavated dens.

**Figure 4:**
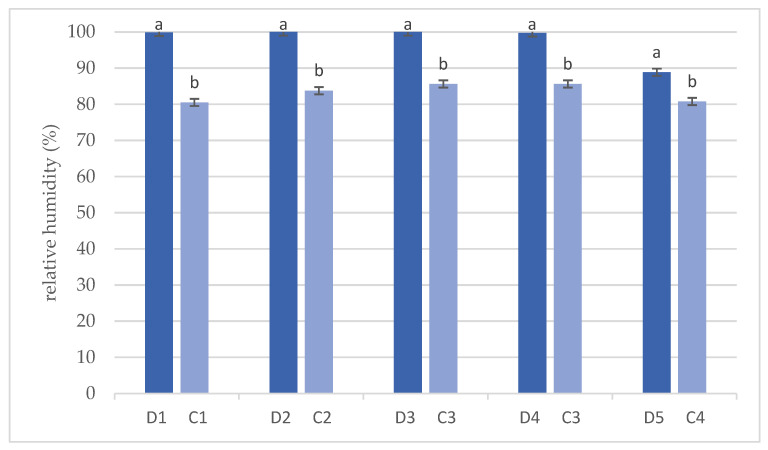
Results of Student’s *t*-test for the relative humidity (%) in dens (D) and corresponding forest stands (C). (Bars represent average ± standard deviation. Values marked with a different letter are significantly different at level *p* < 0.05).

Figure 4 shows the results of the Student’s *t*-test for relative humidity in the dens and corresponding forest stands. The relative humidity was higher in all dens (*p* < 0.0001) than in the forest stands.

**Figure 5:**
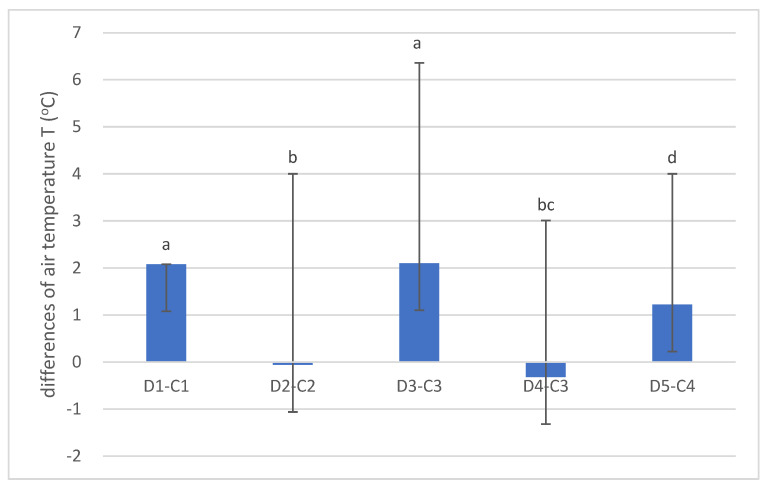
Differences in air temperature (°C) differences between dens (D) and corresponding forest stands (C). (Values marked with a different letter are significantly different at level *p* < 0.05).

The differences between the average daily air temperatures in the den and the forest stand are shown in Figure 5. No significant differences in daily temperature differences were found between dens 1 and 3 (long dens with chambers) and dens 2 and 4 (dens with short tunnels). Daily temperatures differed (*p* < 0.05) between the other dens. Dens 1 and 3 and the corresponding control forest stands showed the greatest temperature difference, 2.08 and 2.1 °C, respectively. Dens 2 and 4 showed a negative temperature difference, −0.05 and −0.31 °C, respectively. It can be assumed that this is due to the high entrance of den 2 and the wide entrance of den 4. In the case of den 2, results must be interpreted cautiously, as the entrance and tunnel of the den face north, while the chamber is located at the end of the tunnel, at an angle of almost 90° to the right and thus protected from the influence of the northern exposure. This den has a narrow but high entrance and a narrow, high tunnel. The measuring device was located at the end of the tunnel. Den 4 has a low but wide entrance. It is a short den without a chamber, which cannot create a specific and favorable microclimate due to its morphological characteristics.

**Figure 6:**
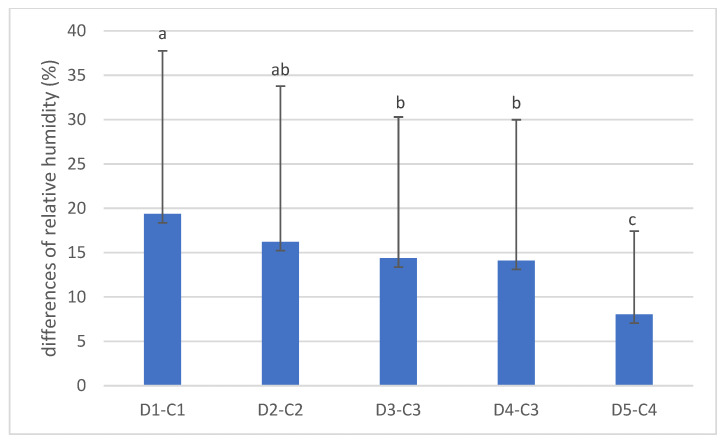
Differences in relative humidity (%) between dens (D) and corresponding forest stands (C). (Values marked with a different letter are significantly different at level *p* < 0.05).

The differences between the average daily relative humidity in the den and the forest stand are shown in Figure 6. There were no differences (*p* > 0.05) between the relative humidity differences between dens 1 and 2, dens 2 and 3, dens 2 and 4, and dens 3 and 4. The largest difference was 19.37% in den 1 and the corresponding forest stand, and the smallest was 8.05% in den 5. These results can be attributed to the den type, i.e., den 1 is a den with a long tunnel with a chamber and a small air volume, while den 5 is a den with a large opening, a short tunnel and no chamber (rock shelter).

### 3.2. Correlation of Microclimatic Elements Between Dens and Forest Stands

The correlation coefficients between the air temperature of the dens and the corresponding forest stands are shown in Table 3. The correlation coefficients were weak for large dens (den 1 and 3), medium to strong for small dens (den 2 and 4), and complete for the rock shelter den (den 5). The correlation coefficients between the air temperature in the dens and the forest stands confirmed that the morphological characteristics of the dens determine the microclimatic conditions, especially the air temperature in the dens. When the air temperature in the forest stands increased or decreased, the air temperature in the dens increased or decreased significantly. However, dens with a high or wide entrance, a short tunnel, and rock shelters were more affected by the change in air temperature in the forest stand.

Table 4 also presents the correlation coefficients of the relative humidity of the dens and the corresponding forest stands. The relative humidity of the dens was not affected by the humidity of the forest stands. There was no significant correlation between the relative humidity in the dens and the corresponding control plots in the forest, except for den 5. There was a strong positive correlation (*p* < 0.0001) between the relative humidity in den 5 and the corresponding forest stand. Den 5 was very specific in terms of its dimensions, as it had a large opening where the dynamics and the change in relative humidity were the same between the den and the forest stand. The large dimensions of the entrance to the den also allowed the entry of a large amount of relative humidity from the forest stand. It should be noted that this den had no chamber and only a very small tunnel. It is more of a shelter in a rock.

Table 4 shows the effects of brown bear den size (small, big) and status (active/inactive) on den temperature and relative humidity. The analysis showed that big and inactive dens had a higher temperature (*p* = 0.0001) than small and active dens. The relative humidity in big and active dens was lower (*p* = 0.001) than in small and inactive dens. It seems that the presence of a bear in the den is not crucial for the temperature conditions in the den. However, it should be considered that in the present study, no camera traps were used to monitor how long and during which period bears were present in active dens. Furthermore, the correlation coefficients presented in Table 3 show that the microclimatic conditions in the forest stands had a major influence on the conditions in the dens. This influence was highly dependent on the morphological characteristics of the dens. Although small and active dens would be expected to have higher temperatures, the den’s exposure and morphology must be considered when interpreting the results. For example, the straight tunnel and northern exposure in den 2 (small and active) probably contributed to lower temperatures. It also seems important to remember that the measuring devices were placed in the ´tunnel of the den. This may have contributed to lower temperatures in the case of den 2 (straight tunnel and northern exposure) regardless of a bear’s presence. It should be considered that this den was in a mesophilic forest stand (beech–fir forest). The higher temperatures in the inactive dens can be attributed to their morphology, exposure, and the microclimate in the forest stand. For example, inactive dens 1 and 5 had the highest average temperatures (Table 1), which can be attributed to thermophilic forest stands (black hornbeam forest). In the case of den 5, the entrance was large and south-facing, so the measuring device was probably more exposed to direct sunlight during the measurement period. Den 1 was the only inactive den without a negative minimum temperature, which can be attributed to the thermophilic forest and the narrow, elongated tunnel facing east. Lower relative humidity in big dens (1 and 5) can probably be attributed to the morphology of den 5, in which the lowest average relative humidity was measured (Table 1). A short tunnel, large entrance, and southern exposure can be considered as the main factors for such results. The same reasons could be responsible for higher relative humidity in active dens. Namely, active den 3 had the highest volume and floor area, and a long tunnel with a chamber at the end.

## 4. Conclusions

The microclimate conditions of the forest stand affect the temperature and relative humidity in the dens, causing higher differences in temperature than in relative humidity. It seems that bear presence is not crucial for temperature or humidity conditions in the den. Big and inactive dens seem to have warmer and less humid conditions than small and active dens. However, detailed morphology characteristics of dens should be considered when interpreting a den’s microclimate conditions, including entrance size and exposure, presence of tunnel and chamber, and length of the tunnel. To confirm the obtained results and to obtain more detailed insight into bear denning behavior, further research including camera traps and a larger number of dens should be undertaken.

## Figures and Tables

**Table 1:** Description of den size, status, exposure, altitude, and forest ecosystem in the denning area.

Den Number	Size (m^3^)	Status ^1^	Exposure (Azimuth °)	Altitude (m)	Forest Ecosystem
1	Small	No	East	989	Black hornbeam
2	Small	Yes	North	979	Beech–fir
3	Big	Yes	East	940	Beech–fir
4	Small	No	East	975	Beech–fir
5	Big	No	South	950	Black hornbeam

^1^ No = no bear activity/no hibernation; Yes = traces of bear activity/hibernation. Small = volume of the den < 5 m^3^; Big = volume of the den > 5 m^3^.

**Table 2:** Descriptive statistics of air temperature (°C) and relative humidity (%) in dens and control forest stands.

Den/Control	Air Temperature (°C)		Relative Humidity (%)	
	Average ± sd	Min.–Max.	Average ± sd	Min.–Max.
Den 1	7.89 ± 1.13	5.99–11.3	99.89 ± 0.46	96.48–100
Den 2	5.23 ± 1.73	1.17–10.37	99.99 ± 0.04	99.61–100
Den 3	6.78 ± 1.28	4.03–10.55	99.99 ± 0.02	99.83–100
Den 4	4.36 ± 2.43	−2.88–10.31	99.74 ± 1.08	92.74–100
Den 5	7.08 ± 2.58	−0.38–13.62	88.85 ± 7.96	64.68–100
Control 1	5.81 ± 4.85	−8.51–18.80	80.52 ± 18.34	24.62–100
Control 2	5.29 ± 4.72	−8.61–18.18	83.77 ± 17.56	21.72–100
Control 3 ^1^	4.67 ± 4.67	−8.98–16.77	85.62 ± 15.93	32.47–100
Control 5	5.86 ± 5.07	−7.62–17.33	80. 79± 13.23	45.36–100

^1^ Control 3 was used for den 3 and den 4 due to their vicinity; sd = standard deviation; Min = minimum; Max = maximum.

**Table 3:** Correlation of air temperature (°C) and relative humidity (%) in dens (D) and corresponding forest stands.

Den/Control	Air Temperature (°C)			
	Control 1	Control 2	Control 3	Control 5
den1	0.221 0.003	0.244 0.001	0.268 0.0003	−0.343 <0.0001
den 2	0.515 <0.0001	0.537 <0.0001	0.549 <0.0001	−0.194 0.013
den 3	0.396 <0.0001	0.422 <0.0001	0.439 <0.0001	−0.261 0.001
den 4	0.705 <0.0001	0.721 <0.0001	0.731 <0.0001	−0.045 0.572
den 5	0.205 0.009	0.203 0.01	0.182 0.020	0.942 <0.0001
**Den/Control**	**Relative Humidity (%)**			
	**Control 1**	**Control 2**	**Control 3**	**Control 5**
den 1	−0.079 0.285	−0.133 0.073	−0.149 0.045	0.071 0.371
den 2	0.096 0.195	0.126 0.091	0.037 0.623	−0.060 0.446
den 3	0.019 0.804	0.036 0.631	−0.049 0.504	−0.049 0.537
den 4	0.113 0.129	0.111 0.137	0.074 0.318	−0.021 0.787
den 5	−0.133 0.0908	−0.144 0.067	−0.125 0.111	0.713 <0.0001

**Table 4:** Effect of brown bear den size and status on den’s microclimate parameters.

Microclimate Parameter	Size		Status		*p* Value		
	Small	Big	Active	Inactive	Size	Status	Size*Status
Temperature (°C)	5.83 ± 2.37	6.92 ± 2.00	5.46 ± 2.12	7.51 ± 1.99	0.0001	0.0001	0.0001
Relative humidity (%)	99.88 ± 0.68	94.73 ± 7.80	99.91 ± 0.63	94.68 ± 7.77	0.001	0.001	0.001

Small = den volume < 5 m^3^; Big = den volume > 5 m^3^; Active = evidence of bear activity; Inactive = no evidence of bear activity; Size*Status = effect of interaction between den’s size and status.

## Data Availability

Dataset available on request from the authors.
